# Tenascin-C Enhances Pancreatic Cancer Cell Growth and Motility and Affects Cell Adhesion through Activation of the Integrin Pathway

**DOI:** 10.1371/journal.pone.0021684

**Published:** 2011-06-29

**Authors:** Igor Paron, Sonja Berchtold, Julia Vörös, Madhavi Shamarla, Mert Erkan, Heinz Höfler, Irene Esposito

**Affiliations:** 1 Institute of Pathology, Helmholtz Zentrum München, German Research Center for Environmental Health, Neuherberg, Germany; 2 Institute of Pathology, Technische Universität München, Munich, Germany; 3 Department of General Surgery, Klinikum rechts der Isar, Technische Universität München, Munich, Germany; Northwestern University, United States of America

## Abstract

**Background:**

Pancreatic cancer (PDAC) is characterized by an abundant fibrous tissue rich in Tenascin-C (TNC), a large ECM glycoprotein mainly synthesized by pancreatic stellate cells (PSCs). In human pancreatic tissues, TNC expression increases in the progression from low-grade precursor lesions to invasive cancer. Aim of this study was the functional characterization of the effects of TNC on biologic relevant properties of pancreatic cancer cells.

**Methods:**

Proliferation, migration and adhesion assays were performed on pancreatic cancer cell lines treated with TNC or grown on a TNC-rich matrix. Stable transfectants expressing the *large* TNC splice variant were generated to test the effects of endogenous TNC. TNC-dependent integrin signaling was investigated by immunoblotting, immunofluorescence and pharmacological inhibition.

**Results:**

Endogenous TNC promoted pancreatic cancer cell growth and migration. A TNC-rich matrix also enhanced migration as well as the adhesion to the uncoated growth surface of poorly differentiated cell lines. In contrast, adhesion to fibronectin was significantly decreased in the presence of TNC. The effects of TNC on cell adhesion were paralleled by changes in the activation state of paxillin and Akt.

**Conclusion:**

TNC affects proliferation, migration and adhesion of poorly differentiated pancreatic cancer cell lines and might therefore play a role in PDAC spreading and metastasis *in vivo*.

## Introduction

Pancreatic ductal adenocarcinoma (PDAC) represents 85–90% of all pancreatic neoplasms. In 2010 43,140 people in the US were estimated to be diagnosed with pancreatic cancer and 36,800 to die of this lethal disease, making PDAC the fourth most common cause of tumor-related mortality in spite of an incidence of only 3% of total cases [1]. PDAC survival rates improved only marginally in the last 30 years and PDAC patients still have a very poor prognosis, with an overall 5-year survival rate below 5% and a median survival rate that ranges from 2 months in patients with metastatic disease to 8 months in patients with non-metastatic disease at the time of diagnosis [2]. PDAC outcomes did not change much in the last several years mainly because of late diagnosis. PDAC as localized and regional disease is mainly asymptomatic and tools for an early detection are still missing. Once diagnosed, pancreatic cancer is often refractory to any chemotherapy and radiotherapy treatment, and many clinical trials have failed to demonstrate a significant improvement in overall survival during the last decade [3]. Therefore, a better comprehension of the mechanisms involved in PDAC development, maintenance and spreading is needed to develop new targeted therapies for the treatment of this nowadays still fatal disease.

A characteristic feature of PDAC is the so-called “desmoplastic” reaction, an abundant fibrous tissue that surrounds cancer cells and mainly consists of blood vessels and stromal cells spread in a scaffold of extracellular matrix (ECM). The development of the desmoplastic reaction is mainly due to pancreatic stellate cells (PSCs). PSCs are stromal cells which, after conversion from a quiescent into an active myofibroblast-like phenotype, secrete ECM proteins and matrix degrading enzymes, thus establishing an environment that strongly promotes cancer progression and, at the same time, imposes a barrier to drug delivery [4]–[7].

Tenascin-C (TNC) is a large ECM glycoprotein composed of six monomers linked at their N-termini with disulfide bonds to form a 1080–1500 kDa hexamer. Each monomer consists of different structural motifs arranged in a linear order, including between 8 and 15 fibronectin type III (FN-III)-like repeats [8], [9]. The alternative splicing of the FN-III-like repeats is able to modulate the biological function of TNC by modifying its interaction with other ECM proteins, like fibronectin (FN), or with cell surface receptors, like integrins or annexin II, and by conferring sometimes opposite roles to TNC in cell spreading, adhesion and proliferation [10], [11].

TNC is mainly expressed during embryonic development. In adults, TNC has a limited pattern of expression (in the basement membrane of the skin, in the ducts of the salivary glands, in the colon mucosa and in the vessel walls of different organs), but protein levels rise dramatically under various physiological and pathological conditions, such as tissue remodeling, neovascularisation and inflammation [12], [13]. Moreover, most solid tumors express high levels of TNC. TNC is able to influence cancer growth by affecting cell adhesion and motility in a way that can promote invasion and metastasis [14] and by influencing the cellular expression of tumor suppressor genes, oncogenes and genes involved in the maintenance of genome stability [15]. In the normal pancreas, TNC is expressed in the muscle wall of blood vessels and in the stromal tissue around the interlobular ducts. TNC expression is up-regulated in acute and chronic pancreatitis [16], and increases in the progression from low-grade precursor lesions (pancreatic intraepithelial neoplasia, PanIN) to PDAC [17]. In PDAC TNC is exclusively expressed in the stroma around the neoplastic glands [16], [17]. Up-regulation of TNC in cancer progression seems to involve specifically the *large* splice variant, as the largest TNC transcript, corresponding to the unspliced form of TNC, is found in pancreatic cancer and in chronic pancreatitis, but not in the normal pancreas [17]. PSCs have been shown to be the main source of TNC *in vivo,* whereas PDAC cells did not show any expression of TNC either by immunohistochemistry or immunoblotting. However, low levels of TNC mRNA were found in pancreatic cancer cell lines by real-time quantitative PCR [17].

In this study, we performed an extensive analysis of the effects of exogenous TNC on pancreatic cancer cell functions and we investigated the effect of endogenous TNC overexpression in the pancreatic cancer cell line PANC-1. Our results point to a main role of TNC in the regulation of the interactions between epithelial cells and ECM in the progression of pancreatic cancer.

## Results

### Effects of exogenous TNC on pancreatic cancer cell growth

**Figure 1:**
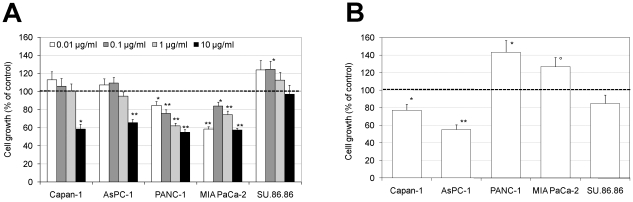
Effects of exogenous TNC on pancreatic cancer cell growth. (A) Cells were grown for 72 hours in serum free medium containing different concentrations of TNC (0.01, 0.1, 1 and 10 µg/ml). (B) Cells were grown for 72 hours in serum free medium onto TNC coated plates (1 µg/cm^2^). Growth was determined with MTT assay. Data are calculated as mean +/− s.e.m. of three experiments and are expressed as percentage compared to the untreated controls (°p<0.05, *p<0.01, **p<0.001, Students two-tailed t test).

At first, it was tested if TNC added to the culture medium at different concentrations could affect cell viability. An increase in the number of viable Capan-1, AsPC-1 and SU.86.86 cells (up to 25%) at TNC concentration of 0.01–0.1 µg/ml was observed, as measured with the MTT assay after 72 hours of growth in serum free medium (Fig. 1A). Growth of AsPC-1 and Capan-1 was inhibited at the highest TNC concentration (10 µg/ml, 41 and 34% decrease, respectively). TNC had an inhibitory effect on the viability of the PANC-1 and MIA PaCa-2 cell lines (Fig. 1A). Since TNC exerts its function as an ECM-protein, cells were grown on a TNC-rich matrix. When seeded on TNC-coated plates and grown up to 72 hours in serum-free medium, PANC-1 and MIA PaCa-2 showed a 44% and 27% increase in viability, respectively, whereas the growth of Capan-1 and AsPC-1 decreased (23% and 45%, respectively) (Fig. 1B).

### Exogenous TNC modulates the motility of pancreatic cancer cell lines

**Figure 2:**
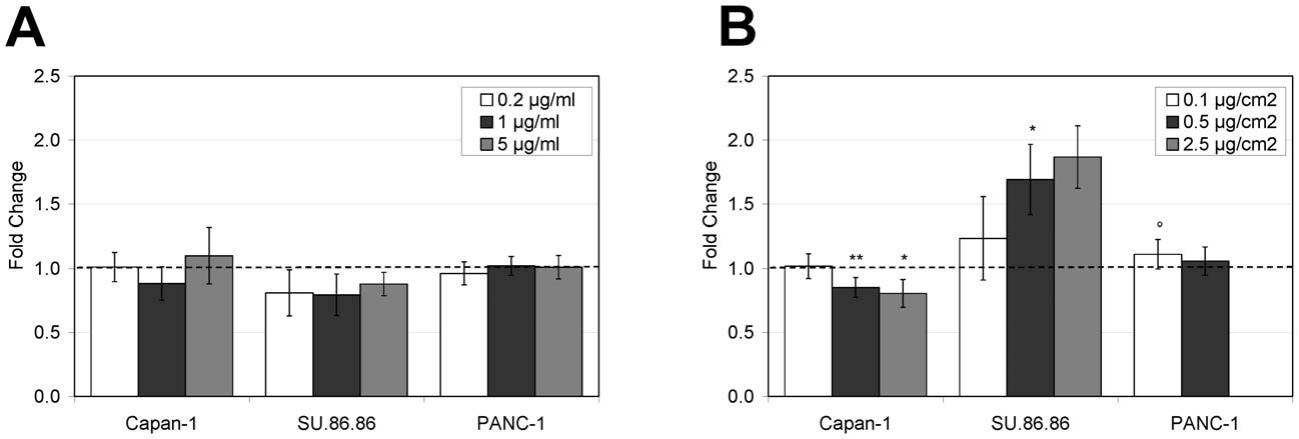
Effect of TNC on the migration of pancreatic cancer cell lines. (A)Cells were plated onto uncoated plates and after 24 hours the monolayer was scraped with a 10 µl pipette tip. Cells were then incubated in serum free medium or in medium with the addition of TNC at different concentrations (0.2 µg/ml, 1 µg/ml and 5 µg/ml) up to 48 hours. (B) Cells were plated onto 24-well plates coated with three different concentrations of TNC (0.1 µg/cm^2^, 0.5 µg/cm^2^ and 2.5 µg/cm^2^) or onto uncoated plates and after 24 hours the monolayer was scraped with a 10 µl pipette tip. Cells were then incubated in serum free medium up to 48 hours. Migration of cells into wounded areas was evaluated counting migrated cells manually and by the TScratch software [36] and a total of 2–8 fields were counted per group in each experiment. Data are calculated as mean +/− s.e.m. and expressed as fold-change compared to the non treated cells (°p<0.05, *p<0.01, **p<0.001, Students two-tailed t test).

In order to test the effect of TNC on the migration of pancreatic cancer cell lines, wound healing assays were performed maintaining the cells in serum free medium in order to minimize cell proliferation. TNC had no effect on the wound closure when added to the growth medium (Fig. 2A). When grown on a TNC-rich matrix, pancreatic cancer cells closed the wound in a dose-dependent way, but each cell line displayed a quite individual response to different concentrations of TNC. In detail, cell migration increased up to 1.7 and 1.1-fold in the SU.86.86 and PANC-1 cell lines, respectively, reaching statistical significance at a TNC concentration of 0.5 µg/cm2 in SU.86.86 cells and of 0.1 µg/cm2 in PANC-1 cells. On the other hand, the wound closure decreased significantly (up to 0.8-fold) in Capan-1 cells with TNC concentrations of 0.5 and 2.5 µg/cm2 (Fig. 2B). A TNC concentration of 2.5 µg/cm2 had toxic effects on the PANC-1 cells, and the wound healing assay could not be performed under this condition.

### Effects of endogenous TNC on cell viability and migration

**Figure 3:**
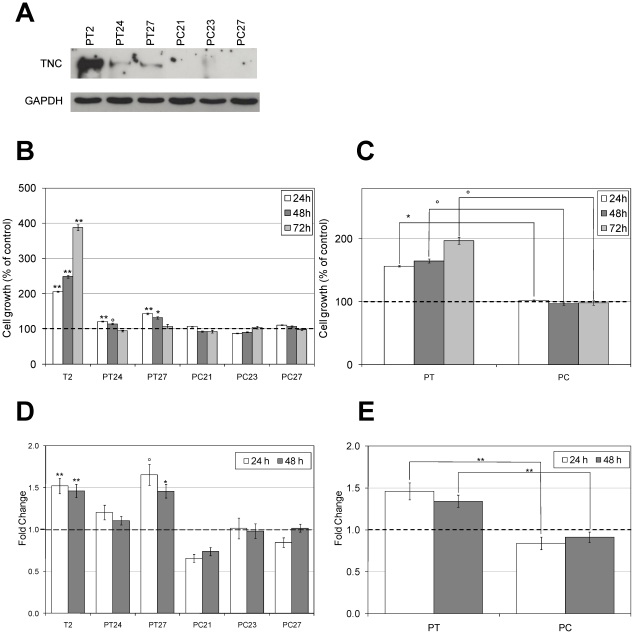
Effects of endogenous TNC on cell proliferation and migration. PANC-1 cells were stably transfected with a vector driving the expression of *large* TNC (PANC-T2, PANC-T24 and PANC-T27 cells) and with the empty vector (PANC-C21, PANC-C23 and PANC-C27 cells). (A) Immunoblotting of precipitated cell culture medium of the transfected PANC-1 cells grown up to 80% confluence. To ensure that a comparable number of transfected cells was the source of secreted TNC, GAPDH expression in whole cell extracts was tested. PANC-T2 cells show the highest levels of secreted TNC, while lower levels are observed in PANC-T24 and PANC-T27. The control clones in comparison do not show any TNC secretion. (B) Cells were grown in complete medium. Growth was determined with MTT assay at different time points (24, 48 and 72 hours). Data are calculated as mean +/− s.e.m. of three experiments and are expressed as percentage compared to the PANC-1 cells (°p<0.05, *p<0.01, **p<0.001, Students two-tailed t test). The proliferation rate is significantly higher for PANC-T2 at all time points (p<0.001), for PANC-T24 at 24 hours (p<0.001) and at 48 hours (p = 0.022) and for PANC-T27 at 24 (p<0.001) and 48 hours (p = 0.009). (C) The proliferation of all the PANC-1 positive clones (PT) compared to the mock transfected cells (PC) is significantly higher for each tested time point (24 hours: p = 0.004, 48 hours: p = 0.012, 72 hours: p = 0.026) (D) Transfected PANC-1 cells were plated, after 24 hours medium was changed with medium containing 0.1% FBS and, after overnight incubation, the monolayer was scraped with a 10 µl pipette tip. Data are calculated as described in B. In comparison to the non transfected PANC-1 cells, migration is significantly faster for PANC-T2 (p<0.001 at both time points) and for PANC-T27 (p = 0.042 at 24 hours; p = 0.009 at 48 hours). (E) All together, the PANC-1 positive clones (PT) migrate significantly faster compared to the mock transfected cells (PC) at both time points (p<0.001).

To give further support to the observed promoting effects of TNC on pancreatic cancer cell growth and motility in the coating procedure, PANC-1 cells were used to generate stable transfectants expressing the *large* TNC splice variant. Since TNC physiologically acts as an extracellular protein, positive clones were further selected on the basis of their capability to secrete TNC in the culture medium. As shown in figure 3A, the expression levels of secreted TNC were quite different among positive clones. Different expression levels reflected on some of the biological activities of TNC, such as its capability to affect cell viability. In fact, the number of viable cells, as measured with the MTT method after 24, 48 and 72 hours of growth in complete medium, was higher in the TNC-positive clones compared both to the non transfected and to the mock transfected PANC-1 cells (Fig. 3B and C). The strongest effect (compared to the non transfected cells) was observed in the T2 clone, which also showed the highest expression levels of secreted TNC (Fig. 3A and B). In detail, in comparison with non transfected cells, PANC-T2 cells showed an increase in cell viability of 2.06-fold +/−0.01 after 24 hours, of 2.49-fold +/−0.04 after 48 hours and of 3.88+/−0.09 after 72 hours. In PANC-T24 the increase was of 1.21-fold +/−0.01 after 24 hours and of 1.14-fold +/−0.02 after 48 hours, and in PANC-T27 of 1.43-fold +/−0.02 after 24 hours, of 1.32-fold +/−0.03 after 48 hours and 1.08-fold +/−0.05 after 72 hours. The cell viability rates of PANC-1 cells overexpressing TNC compared to the mock transfected cells were 55% higher at 24 hours, 68% higher at 48 hours and 99% higher at 72 hours (Fig. 3C).

Cell migration on the other hand was not influenced by the expression levels of secreted TNC. Stable transfected PANC-T2, PANC-T24 and PANC-T27 cells closed the wound faster than non transfected and mock transfected cells. When compared to the non transfected PANC-1 cells, this effect was significant at 24 hours (1.52-fold +/−0.09) and at 48 hours (1.46-fold +/−0.08) for PANC-T2 and at 24 (1.66-fold +/−0.13) and 48 hours (1.46-fold +/−0.08) for PANC-T27 (Fig. 3D). All positive clones together had a higher migration rate in comparison to the mock transfected clones (62% at 24 hours and 43% at 48 hours, Fig. 3E).

### Exogenous TNC stimulates cell adhesion on uncoated plates and reduces cell spreading on FN

**Figure 4:**
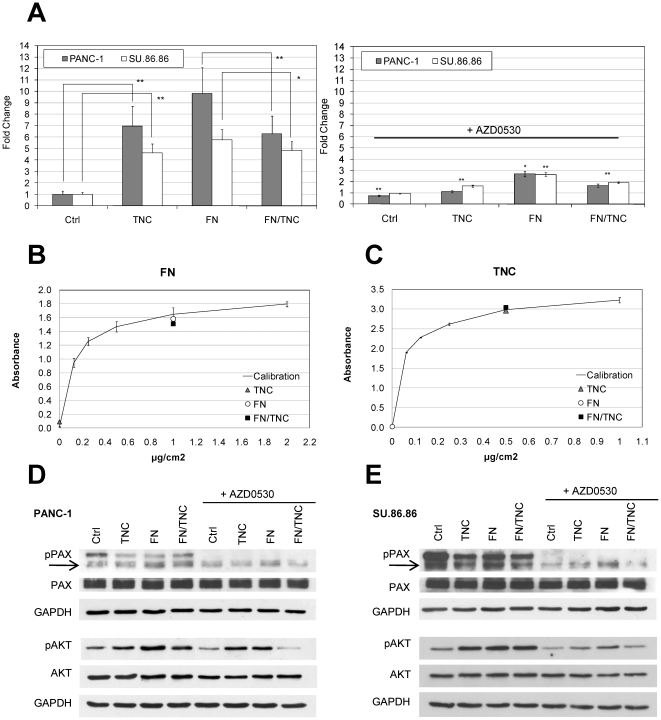
Effect of TNC, FN, and FN/TNC coating on the adhesion of PANC-1 and SU.86.86 cell lines. (A) Cells were plated in medium containing 1% FBS, incubated for 3 hours and fixed. For pharmacological inhibition, cells were incubated with AZD0530 2 µM for 15 min before plating. Left panel: TNC enhances cell adhesion compared to uncoated plates and decreases cell attachment to FN. Right panel: When treated with AZD0530, cells displayed a reduced adhesion compared to the non treated cells at all conditions tested. Data are calculated as mean +/− s.e.m of two experiments and expressed as fold-change compared to the control (Ctrl) (left panel) or to the untreated cells (right panel) (*p<0.01, **p<0.001, ANOVA test). (B, C) 96-well plates were coated with TNC (0.5 µg/cm^2^), FN (1 µg/cm^2^) or both proteins simultaneously (FN/TNC) and ELISA was performed as described in Methods. The substrate binding activity of TNC or FN is not affected when both proteins areused to coat the plastic surface. (D, E) PANC-1 (D) and SU.86.86 cells (E) were plated for 45 min before total protein extraction was performed. Phosphorylation of paxillin at Tyr 118 (pPAX) and of Akt at Ser 473 (pAKT), and total paxillin and Akt expression levels were investigated by immunoblotting using specific antibodies and after treating the cells with the Src kinase inhibitor AZD0530. GAPDH detection was used to confirm equal protein loading.

As cell adhesion together with migration and invasiveness is a critical step involved in cancer spreading and metastasis, the adhesion of pancreatic cancer cells on TNC was further investigated. Since TNC overexpression correlates with poor tumor differentiation *in vivo* [16], we focused our study on the poorly differentiated cell lines PANC-1 and SU.86.86 [18], [19]. TNC coating strongly enhanced the adhesion of both cell lines (PANC-1: 7.0-fold, p<0.001; SU.86.86: 4.6-fold, p<0.001) as estimated by crystal violet absorbance spectroscopy of attached cells (Fig. 4A, left panel). In the tissue context, cells interact with TNC in combination with other ECM proteins. Among these, FN is often co-expressed with TNC [20]. Therefore, in order to test if TNC modulates cell adhesion in the presence of FN, PANC-1 and SU.86.86 cells were plated on a mixed substrate of FN and TNC. TNC presence determined a significant decrease in the attachment of both cell lines to FN at 3 hours after plating (PANC-1: 1.6-fold, p<0.001; SU.86.86: 1.2 fold, p = 0.003) (Fig. 4A, left panel). To test if this effect could be due to substrate competition between the two proteins when used together to coat the plastic surface, the substrate binding efficiency of the composite matrix was investigated by ELISA. The binding efficiency of FN or TNC alone was not affected when the plates were coated with both proteins simultaneously, as shown in figure 4B and C. Cell viability was also not significantly affected when cells were grown onto FN/TNC coated plates compared with FN coated plates (MTT assay, data not shown). Taken together, these results show that TNC interferes with cell adhesion on FN, but it has an opposite effect on uncoated plates.

### TNC and FN influence paxillin and Akt activation by phosphorylation

In order to investigate the pathways involved in the adhesive behavior of pancreatic cancer cells on TNC and/or on FN substrates, the phosphorylation level of paxillin at Tyr 118, an early step in integrin mediated signaling, as well as the phosphorylation level of Akt at Ser 473 and the expression levels of vinculin, a protein involved in the connection of focal adhesions to the actin cytoskeleton, were analyzed by immunoblotting.

As shown in Fig. 4, TNC and FN had a slight enhancing effect on the phosphorylation state of Aktin the PANC-1 and SU.86.86 cell lines during the first steps of cell adhesion (45 min). This effect was not evident anymore after 24 hours and at later time points (not shown). As for cell adhesion, TNC seemed to have opposite effects depending on the experimental set up. In fact phospho-Akt was slightly up-regulated when comparing TNC coated plates *vs* uncoated plates and down-regulated when comparing FN/TNC coated plates *vs* FN coated plates. The same trend could be seen when measuring paxillin activation, while vinculin levels were not affected either by TNC or FN (not shown). The Src kinase inhibitor AZD0530 (Saracatinib), which inhibits the phosphorylation of paxillin and Akt, was used to confirm that the observed effects on cell adhesion were actually mediated by molecules of the integrin signaling pathway. In adhesion experiments, after incubation with AZD0530 an inhibition of paxillin and Akt phosphorylation was observed (Fig. 4D and E). This effect was accompanied by a significant decrease in PANC-1 and SU.86.86 adhesion at all conditions tested (PANC-1 FN: p = 0.004; all other conditions p<0.001), with the exception of SU.86.86 adhesion to uncoated plates (Fig. 4A, right panel).

**Figure 5:**
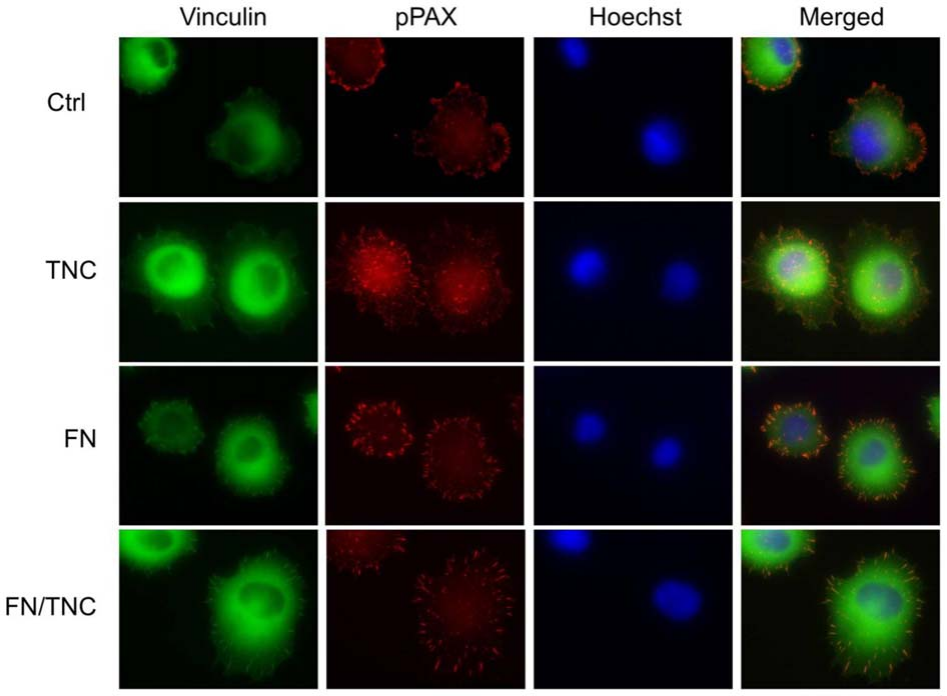
Cellular distribution of phospho-paxillin and vinculin proteins on coated surfaces. PANC-1 cells were let adhere to uncoated or TNC, FN and FN/TNC coated coverslips for 45 min, gently washed, fixed and stained using fluorescent secondary antibodies. Focal adhesion plaques were evidenced by co-localization of vinculin (green) and phospho-paxillin (pPAX, red). Cell nuclei were counterstained with Hoechst 33342.

Next, TNC effect on focal adhesion assembly was investigated on a morphological level by immunofluorescence. As shown in Fig. 5, PANC-1 cells grown for 45 min on a TNC coated surface showed a more diffuse co-localization pattern of vinculin and phospho-paxillin than that observed on the uncoated surface, where focal adhesions appeared more elongated and mainly concentrated at the periphery of the spreading cell. On a FN matrix, only slightly noticeable differences in phospho-paxillin and vinculin distribution between cells adhered on FN and FN/TNC were observed. In both cases, phospho-paxillin/vinculin areas were mainly restricted to the periphery of the adhering cells, with longer and more dispersed adhesion sites in the presence of TNC.

## Discussion

PDAC is characterized by a prominent increase in the connective tissue that surrounds cancer cells. Major contributors to this so-called “desmoplastic” reaction are PSCs, a subpopulation of pancreatic cells that, once activated by growth factors, cytokines and oxidative stress, secrete excess amounts of ECM proteins and soluble mediators establishing a cross-talk with tumor cells. Several lines of evidence suggest that the desmoplastic microenvironment plays a key role in regulating the rapid growth and invasion of pancreatic cancer, angiogenesis and resistance to chemotherapy [4]–[7], [21]–[26].

TNC is a large stromal ECM protein that is up-regulated in the progression from PanINs to PDAC [17] and its expression has been correlated with a poorly differentiated phenotype of PDAC [16]. Moreover, TNC expression has been correlated with a poor prognosis of patients affected by lung and brain cancer [21], [27]–[29], whereas no correlation between expression levels and prognosis has been found in pancreatic cancer [16]. TNC is able to interact with several ECM proteins (such as FN, perlecan, aggrecan, versican and brevican) and many cell-surface receptors (including integrins α2β1, α7β1, α9β1, αvβ3, annexin II, EGFR and syndecan 4), modulating cellular signaling and influencing cell migration and proliferation [11]. Additionally, in the adult organism TNC has adhesive and anti-adhesive properties and is expressed during physiological and pathological conditions where cell migration and tissue remodeling is involved, like in the wound healing process or during tumorigenesis and metastasis [11]. However, the molecular mechanisms by which TNC influences cancer progression are still largely unknown. To address this question in PDAC, we have investigated how TNC affects biologic relevant properties of pancreatic cancer cells.

The results presented here demonstrate that TNC is able to support and promote growth and migration of poorly differentiated pancreatic cancer cell lines. Another interesting observation is that TNC alone shows a pro-adhesive effect on pancreatic cancer cells, in contrast to the reported anti-adhesive effect in the majority of other studied cell lines, like glioblastoma and breast carcinoma cells [22]. This pro-adhesive effect on pancreatic cancer cells is associated with an increase in the phosphorylation state of Akt and to a lesser extent of paxillin. This suggests a mechanism involving integrin-mediated adhesion to TNC, in analogy to what has been previously demonstrated in chondrosarcoma cells, where an increase of Ser 473 phosphorylation of Akt in cells adhering to TNC promotes cell survival in serum deprived medium [23]. However, the effects of TNC on pancreatic cancer cell viability and proliferation were quite heterogeneous and an inhibition of the growth was observed in some cell lines, mostly at the highest concentrations of TNC. This result is not surprising, due to the heterogeneity of PDAC cell lines concerning their origin and differentiation [19], [20] and to the pleiotropic and often opposite effects of TNC depending on the cell and tissue context. *In vitro* studies often report contradictory results, with TNC both stimulating [24] and inhibiting [25] cell growth and promoting both cell adhesion and detachment [26]. Moreover, opposite adhesive and migratory responses to TNC in the same glioma cell line depending on the integrin receptor involved [30] and different adhesive responses mediated by the same integrin in different cell lines [31] have been described. Interestingly, on a FN coated surface TNC decreases pancreatic cancer cell adhesion and phospho-paxillin and phospho-Akt levels. This effect on a mixed TNC-FN matrix, which closely resembles the *in vivo* situation, where TNC and FN interact in the tumor-associated ECM, has already been described for glioblastoma and breast carcinoma cells [20], [22]. In these tumors, TNC reduces cell spreading on FN interfering with FN binding to the integrin co-receptor syndecan-4, thus compromising focal contact and stress fiber formation [29]. Since the substrate binding efficiency of FN was not affected by TNC in our study, as assessed by ELISA, a similar mechanism of competition for cell surface receptors can be hypothesized in PDAC cells. Due to this effect, activation of paxillin and focal adhesion kinase (FAK) and, consequently, actin stress fiber and focal contact formation as well as full cell spreading is inhibited, with an overall enhancement in cell proliferation [22]. Weak binding to FN usually correlates with an enhanced proliferation in many tumor cells [32]. In our experimental set up, we did not observe an increase in cell proliferation for PANC-1 and SU.86.86 cells on a mixed substrate of FN and TNC compared to FN alone, probably because the effects of TNC on adhesion were limited to short times (3 hours) and focal adhesion assembly in the FN-adherent cells was only slightly altered by TNC, as observed by immunofluorescence. These observations suggest that the mechanisms by which TNC influences cancer cell adhesion and proliferation on a FN substrate are not general but strictly dependent on the specific cell line and on the experimental set up used.

It is known that the ability of cancer cells to interact with extracellular matrix proteins, like FN, is crucial for cell invasion and migration. Our data show that the number of pancreatic cancer cells that adhere to FN at short times is decreased in the presence of TNC but is still high and in the range of the number of cells adhering to TNC alone. TNC may therefore act as a modulator of early migratory events, which include substrate sensing and adhesion, and may promote cell migration by reducing cell adhesiveness to FN, thus inducing a more dynamic interaction between the tumor cell and the ECM components. Further work is needed to understand how TNC affects the adhesion process during pancreatic cancer cell interaction with FN, which cell receptors are involved and which additional intracellular pathways may be altered.

In conclusion, the present data show that TNC affects growth, motility and adhesion of pancreatic cancer cells, these effects being divergent depending on cell differentiation and on the composition of the extracellular matrix. Therapeutic strategies aiming at disrupting the TNC-rich matrix might be therefore helpful in contrasting the extraordinary aggressiveness of PDAC.

## Materials and Methods

### Maintenance of cell lines

Pancreatic cells of lines Capan-1, AsPC-1, PANC-1, MIA PaCa-2 and SU.86.86 [18], [19], [33] (American Type Culture Collection, ATCC) were maintained in high glucose DMEM media containing 10% fetal bovine serum (FBS), and 1 X Penicilline/Streptomicine (Invitrogen, Carlsbad, CA) at 37°C in a humified atmosphere with 5% CO2. Cells were tested for mycoplasma contamination by polymerase chain reaction (TAKARA, Shiga, Japan).

### Generation of stable clones overexpressing TNC

The plasmid TNC-L, in which the large, unspliced isoform of TNC is cloned into a pCMV-Script vector (Stratagene, La Jolla, CA, USA), was a generous gift from Dr. JH Pringle (Department of Cancer Studies & Molecular Medicine, University of Leicester School of Medicine, UK) [34]. The complete TNC-L coding sequence was checked by sequencing. Then, the TNC-L plasmid and the empty vector pCMV-Script were first linearized with the restriction enzyme APA-L1 and then transfected into the PANC-1 cancer cells using the FuGENE transfection reagent (Roche Diagnostics, Mannheim, Germany), according to the manufacturer's instructions. After 2 days, cells were subjected to G418 selection at a concentration of 1 mg/ml. After 2 weeks in the presence of G418, several colonies were observed and amplified. Colonies were checked for the presence of the transfected gene by PCR assay using the primers 5-CGTGTACGGTGGGAGGTCTA-3 (on pCMV-Script) and 5-GACACCAGGTTCTCCAGCTC-3 (in the TNC gene). The capability of PCR-positive colonies to secrete TNC was confirmed by Western blotting. The positive clones PANC-T2, PANC-T24, PANC-T27 and the control (mock transfected) clones PANC-C21, PANC-C23, PANC-C27 were selected for the following experiments.

### TNC and FN coating

TNC was purchased from Millipore (Millipore GmbH, Schwalbach, Germany) and FN from Biochrom (Berlin, Germany). For 96-well plates (viability and adhesion), coating was performed with TNC at a final concentration of 1 µg/cm2. For 24-well plates (wound healing assays) TNC concentrations of 0.1 µg/cm2, 0.5 µg/cm2 and 2.5 µg/cm2 were used. For 6-well plates (immunoblotting) a concentration of 0.5 µg/cm2 was used. FN coating was performed with a final concentration of 2 µg/cm2 for 96-well plates and 1 µg/cm2 for 24- and 6-well plates. Coating proteins were allowed to be adsorbed overnight at 4°C, wells were then washed with PBS to remove unbound proteins and blocked for 30 min at 37°C by the addition of 0.2% heat-denatured (85°C for 12 min) BSA in PBS. Plates were finally washed twice with sterile PBS and sterilized by UV exposure for 20 min. Coverslips used in immunofluorescence experiments were placed into 24-multi-well plates and coated in the same way. Mixed substrates of TNC and FN were prepared by coating with both proteins simultaneously. Uncoated plates, blocked with BSA, are referred in this manuscript as “uncoated”.

### Growth assay

Cells were seeded into 96-multi-well plates (Nunc GmbH & Co. KG, Langenselbold, Germany) at a density of 20,000/cm2 and in medium containing 10% FBS. After 24 hours the medium was changed with serum free medium containing different concentrations of TNC (0.01, 0.1, 1 and 10 µg/ml). Cells were then grown for 72 hours before 3-(4,5-dimethylthiazol-2-yl)-2,5-diphenyltetrazolium bromide (MTT) assay was performed as previously described [35]. To test the effect of TNC coating, cells from 70–80% confluent cultures were seeded into 96-multi-well plates at a density of 20,000/cm2 in serum-free medium. Cells were then grown up to 72 hours and MTT assay was performed. For experiments with stably transfected PANC-1, cells were seeded into 96-multi-well plates at a density of 10,000/cm2 and in medium containing 10% FBS. Cells were then grown up to 72 hours and MTT assay was performed at different time points (24, 48 and 72 hours).

### Adhesion assay

Cells were trypsinized, resuspended to a concentration of 300,000/ml in medium with 1% FBS and incubated 20 min at 37°C, 5% CO2. For experiment with the Src kinase inhibitor AZD0530 (Saracatinib), cells were further incubated with the drug (2 µM) for 15 min. Then, 50 µl (15,000 cells) were plated onto 96-multi-well plates, whose wells had been filled with 50 µl medium containing 1% FBS and equilibrated at 37°C, 5% CO2 for 1 hour. After 3–24 hours of incubation, medium was aspirated and the wells were washed twice with PBS to remove non-adherent cells. Cells were then fixed with 5% glutaraldehyde in PBS for 20 min and stained for 60 min with a filtered solution of 0.1% (w/v) crystal violet in 200 mM 2-(N-morpholino)ethanesulfonic acid (MES), pH 6.0. Cells were washed four times with water, air-dried and lysed in 100 µl of 10% (v/v) acetic acid in water. Absorption was measured at 560 nm using an ELISA reader. Six-well plates were used for protein extraction. Both adherent and non-adherent cells were collected after 45 min–72 hours of incubation.

### Wound healing assay

Cells were plated onto 24-multi-well plates and after 24 hours the monolayer was scraped with a 10 µl pipette tip. After three washes with PBS, cells were incubated at 37°C, 5% CO2, in serum free medium with different TNC concentrations (0.2 µg/ml, 1 µg/ml and 5 µg/ml) or without TNC. To test the effect of TNC coating, the same procedure was performed on TNC-coated 24-multi-well plates (0.1 µg/cm2, 0.5 µg/cm2 and 2.5 µg/cm2 TNC, respectively). In both cases, the migration of cells into wounded areas was evaluated at the indicated times by taking images with a Zeiss Axiovert microscope coupled with a digital camera (AxioCam ICc3, Carl Zeiss Vision GmbH, Jena, Germany) and counting migrated cells manually. A total of 2–4 fields were counted per group in each experiment.

For experiments with stably transfected cells, cells were plated onto 24-multi-well plates at a density of 75,000 cells/cm2 and, after 6 hours, medium was changed and cells were maintained overnight in medium containing 0.1% FBS. The monolayer was then scraped with a 10 µl pipette tip. After three washes with PBS, cells were incubated at 37°C, 5% CO2, in medium completed with 0.1% FBS. Images were taken at the indicated times with a Evos XL microscope (AMG - Advanced Microscopy Group, Bothell, WA, USA) and cell migration into wounded areas was evaluated using TScratch software [36]. A total of 4–8 fields were counted per group in each experiment.

### Whole cell extract and supernatant preparation

Culture medium was removed and not attached cells were collected by centrifugation. Cell monolayers were rinsed twice with cold PBS. Both pelleted and still adherent cells were lysed in cold lysis buffer (20 mM Tris, 150 mM NaCl, 1% NP-40, 0.5% sodium deoxycholate, 1 mM EDTA, 1 mM EGTA pH 7.4) supplemented with protease and phosphatase inhibitor cocktails (Roche Diagnostics, Mannheim, Germany). Cell lysates were passed through a 26G needle several times and cellular debris was removed by centrifugation at 15000×g for 10 min at 4°C. The protein concentration in the soluble fractions was determined using the BCA protein assay kit (Thermo Scientific, Schwerte, Germany) and the extracts were aliquoted and stored at −80°C. Transfected cell lines were grown in culture medium for 72 hours until cells reached 80% confluence. 250 µl of the supernatant were then taken and precipitated with 4 vol acetone over night at −20°C. The precipitate was centrifuged 20 min at 13.000 rpm and resuspended in 2X Laemmli buffer.

### Immunoblot analysis

Cell lysates were subjected to SDS-PAGE with 5%, 10% or 12% polyacrylamide gels using the Mini-PROTEAN Tetra cell apparatus (Bio-Rad Laboratories GmbH, Munich, Germany) at 110 V for 1 hour in Tris–glycine buffer (25 mM Tris, 250 mM Glycine, 0.1% SDS, pH 8.3). Resolved proteins were transferred from the gels to nitrocellulose membranes using a Mini Trans-Blot Cell electrophoretic transfer cell (Bio-Rad) at 120 V in transfer buffer (48 mM Tris, 39 mM Glycine, 0.025% SDS, 20% Methanol, pH 8.3). Non-specific antibody binding was blocked by incubation of the membranes in 5% BSA in TTBS buffer (20 mM Tris, 136 mM NaCl, 0.1% Tween20, pH 6,8), for 1 hour at room temperature. The membranes were then incubated overnight at 4°C with antibodies anti-phospho-paxillin (Tyr 118, 1∶1000, GeneTex Inc.), anti-paxillin (1∶500, Cell Signaling), anti-GAPDH (1∶1000, Santa Cruz Biotechnology), anti-vinculin (1∶1000, Sigma-Aldrich), anti-phospho-Akt (Ser 473, 1∶1000, Cell Signaling), anti-Akt (1∶1000, Cell Signaling) diluted in TTBS with 3% BSA. After three washes in TTBS, membranes were then probed for 1 hour at room temperature with horseradish peroxidase-conjugated secondary antibodies (anti-mouse or anti-rabbit-IgG, Sigma-Aldrich) diluted 1∶2000 in TTBS with 5% blotting-grade milk, and then washed again three times with TTBS. All steps were performed under gentle agitation on a rotary shaker. The signals were visualized using the Super Signal West Pico Chemiluminescent Kit (Thermo Scientific, Schwerte, Germany) and by light-sensitive imaging film (Amersham Hyperfilm ECL, GE Healthcare, Munich, Germany).

### Immunofluorescence

Cells adhering to coverslips were washed with PBS, fixed with 4% formaldehyde in PBS buffer for 15 min at RT, washed three times with TBS (50 mM Tris, 138 mM NaCl, 2.7 mM KCl, pH 7,6), permeabilized with Triton X-100 (0.25% v/v in TBS) for 5 min, before being washed twice and blocked for 1 hour in blocking solution (TBS containing 10% goat serum and 0.1% Triton X-100). Then, incubation with antibodies anti-phospho-paxillin (Tyr 118, 1∶100, Santa Cruz Biotechnology, CA) and anti-vinculin (1∶200) was performed in the blocking solution at RT in a humidified chamber overnight. After washing three times with TBS, slides were incubated with Alexa Fluor 488 goat anti-mouse-IgG or Alexa Fluor 647 goat anti-rabbit-IgG (1∶200, Invitrogen GmbH, Karlsruhe, Germany) in blocking solution for 1 hour. Slides were then washed again three times before being counterstained with Hoechst 33342 (0.2 µg/ml in water) for 5 min, briefly washed with TBS, covered with anti-fade mounting medium (Vectashield, Loerrach, Germany) and placed onto microscope slides. Slides were examined under a Zeiss Axiovert fluorescence microscope (Carl Zeiss AG, Germany). Negative controls were performed omitting the primary antibodies.

### ELISA

96-well plates were coated with TNC (0.5 µg/cm2), FN (1 µg/cm2) or both proteins simultaneously (FN/TNC). A calibration curve was generated using FN at a density between 125 ng/cm2 and 2 µg/cm2 and TNC at a density between 62.5 ng/cm2 and 1 µg/cm2 to coat the plate.The amount of FN or TNC on the plate was then quantified by the ELISA assay (IBL International GmbH, Hamburg, Germany) according to the manufacturer's instructions.

### Statistical analysis

Means were compared using the Students t-test or the one-way analysis of variance (ANOVA) with the package S-PLUS (Tibco, Palo Alto, CA). Data are presented as mean ± SEM (standard error of the mean).
